# Investigation of the Putative Relationship Between Copper Transport and the Anticancer Activity of Cisplatin in Ductal Pancreatic Adenocarcinoma

**DOI:** 10.3390/cells14191489

**Published:** 2025-09-24

**Authors:** Alina Doctor, Jonas Schädlich, Sandra Hauser, Jens Pietzsch

**Affiliations:** 1 Department of Radiopharmaceutical and Chemical Biology, Institute of Radiopharmaceutical Cancer Research, Helmholtz-Zentrum Dresden-Rossendorf, Bautzner Landstrasse 400, 01328 Dresden, Germany; a.doctor@hzdr.de (A.D.); j.schaedlich@hzdr.de (J.S.); s.hauser@hzdr.de (S.H.); 2 Faculty of Chemistry and Food Chemistry, School of Science, Technische Universität Dresden, Bergstrasse 66, 01069 Dresden, Germany

**Keywords:** ATPases, cancer cells, co-culture model, copper-64, cuproptosis, experimental radiotracer approach, pancreatic stellate cells, repurposing of drugs, tumor microenvironment

## Abstract

Pancreatic ductal adenocarcinoma (PDAC) is a highly heterogeneous cancer with a severe stromal reaction mediated by pancreatic stellate cells (PSCs), leading to increased resistance to chemotherapy and radiotherapy. Following a repurposing concept, this preclinical study investigates the potential of approved drugs, known to be modulators of cellular copper transport, in combination with cisplatin for therapeutic approaches in PDAC. Two major strategies were pursued: (i) inhibiting copper transporters ATP7A and B with tranilast (TR) and omeprazole (OM) to block the cellular copper and, potentially, also cisplatin efflux, and (ii) using the chelator elesclomol (ES) to elevate intracellular copper and cisplatin levels. Human cell lines PanC-1 (PDAC), HPaSteC (PSC), and their co-culture, as well as the hepatocellular carcinoma cell line HepG2 as a reference model, were used. In addition to an analysis of the expression of copper transport proteins, the dynamics of cellular copper uptake and transport were monitored using a [^64^Cu]CuCl_2_ radiotracer approach. In vitro, all drugs enhanced cellular copper uptake and/or reduced copper efflux. Moreover, all drugs contributed to the enhanced cellular anticancer activity of cisplatin, with ES being the most effective compound. The results suggest that the targeted modulation of copper transport mechanisms may offer novel adjuvant approaches for the treatment of PDAC.

## 1. Introduction

Pancreatic cancer remains a relatively rare but highly lethal malignancy. According to GLOBOCAN 2022, pancreatic cancer ranks twelfth in incidence and seventh in mortality worldwide, with both epidemiological measures currently steadily increasing [1]. In Germany, pancreatic cancer is the tenth most common cancer (both sexes combined), with approximately 20,000 (9956 women, 10,271 men) new cases diagnosed in 2020, and the fourth most frequent cause of cancer death, with the number of deaths amounting to about 19,000 (9474 women, 9448 men) in 2020, reflecting its poor prognosis [2]. Current SEER data indicate that most pancreatic cancer is diagnosed at an advanced stage (approximately 52% metastatic, 29% regional, 11% localized, and 8% unknown), with a five-year survival rate of about 12% (all stages combined) [3]. Pancreatic ductal adenocarcinoma (PDAC) is the most clinically relevant sub-entity. Because PDAC lacks characteristic symptoms, it is a highly deceptive cancer. The median survival time is extremely short, approximately six months after diagnosis. Approximately 80% of pancreatic cancers are inoperable and already have a locally advanced stage or distant metastases at diagnosis, the latter primarily in the liver and lungs. Surgical resection is beneficial for only a small percentage of patients (15–20%) [4,5,6].

PDAC is a highly complex entity due to the limited knowledge surrounding its risk factors and the characteristics of precursor lesions. It is a heterogeneous tumor-stroma entity, with up to 90% of the tumor mass consisting of stromal cells [5,7]. The tumor stroma is primarily composed of activated pancreatic stellate cells (PSCs), which produce extensive amounts of extracellular matrix (ECM) proteins, leading to fibrosis [8,9]. The large proportion of stromal cells also leads to abnormal vascularization and resistance to radiation and chemotherapy [4,8,10,11].

The successful introduction of intensified chemotherapeutic regimens, such as gemcitabine/nab-paclitaxel (gemcitabine-Abraxane^®^, or GA) and FOLFIRINOX/mFOLFIRINOX (infusional 5-fluorouracil, leucovorin, irinotecan, and oxaliplatin, or FFX/mFFX), has led to a significant, though still moderate, improvement in response rates, disease control, and overall survival [12,13,14,15]. In clinical practice, approximately a quarter to a third of PDAC patients in Europe receive the platinum-based chemotherapy following the FOLFIRINOX/mFOLFIRINOX regimen [16,17]. Despite the use of these therapy approaches, which has been associated with an improved median overall survival of about 11–12 months, PDAC is considered particularly chemoresistant [18,19].

Consequently, strategies that enhance the efficacy and effectiveness of platinum-based therapy are a clinical need [20]. In accordance with the concept of drug repurposing, we hypothesized that drugs, which modulate the copper transport system, may possess this potential. This is due to the fact that the cellular copper transport mechanism is similar for copper and platinum, or, here synonymously, platinum-containing compounds [21,22,23]. In addition, it has previously been shown that copper levels [24,25] as well as proteins involved in copper transport [26,27,28] are increased in tumor diseases. The transition metal copper is, because of its ability to accept and donate single electrons, essential as a cofactor for a variety of enzymes [29]. In vivo, Cu(II) is reduced to Cu(I) by binding to metalloreductase STEAP [30]. In aqueous solutions, this oxidation state tends to undergo disproportionation. To prevent the resulting formation of reactive oxygen species, copper(I) ions are bound to proteins or small molecules, including human serum albumin (HSA), ceruloplasmin, and transcuprein [31,32]. The transport of the copper(I)-cation is mediated by CTR1 (high-affinity copper uptake protein 1 and also solute carrier family 31 member 1, SLC31A1) [33]. Copper(I) is intracellularly chelated and transported by ATOX1 (antioxidant 1 copper chaperone) to ATP7A/B (copper-transporting P-type ATPases) [34,35]. ATP7A and B have six metal-binding domains and are located on the trans-Golgi network at basal copper levels of 0.5 µM, where they deliver copper to copper-dependent enzymes. At elevated intracellular copper levels, they can excrete copper from the cell. They use energy from ATP hydrolysis for the excretion of excess copper [31,36,37]. ATP7A is expressed in the majority of tissues, with the exception of the liver. In contrast, ATP7B is predominantly expressed in the liver and kidneys [38]. Although ATP7A and B have 60% similarity in amino acid sequence and are therefore similar in terms of structure, they appear to have distinct roles when co-expressed in the same organ [36]. The results of Katano et al. suggest a greater impact of ATP7A on the initial efflux, and of ATP7B on the terminal efflux of carboplatin [39]. Additionally, copper can be transported via CCS (copper chaperone for superoxide dismutase 1) and COX11/17 (copper chaperone for cytochrome c oxidase) to the respective destinations SOD1 (superoxide dismutase 1) and COX (cytochrome c oxidase). Cisplatin can gain access to the cell via the CTR1 transporter and by passive diffusion [40]. The potential contribution of elesclomol (ES) to cisplatin transport is discussed [41]. Once internalized by the cell, cisplatin exerts its cytotoxic effects by binding to DNA within the nucleus. Prior research has demonstrated the effectiveness of tranilast (TR) in inhibiting ATP7B in ovarian cancer cells [42]. Omeprazole (OM) and ES have been shown to inhibit copper transport by affecting ATP7A in melanoma cells [43] and colorectal cancer cells [44], respectively.

**Figure 1:**
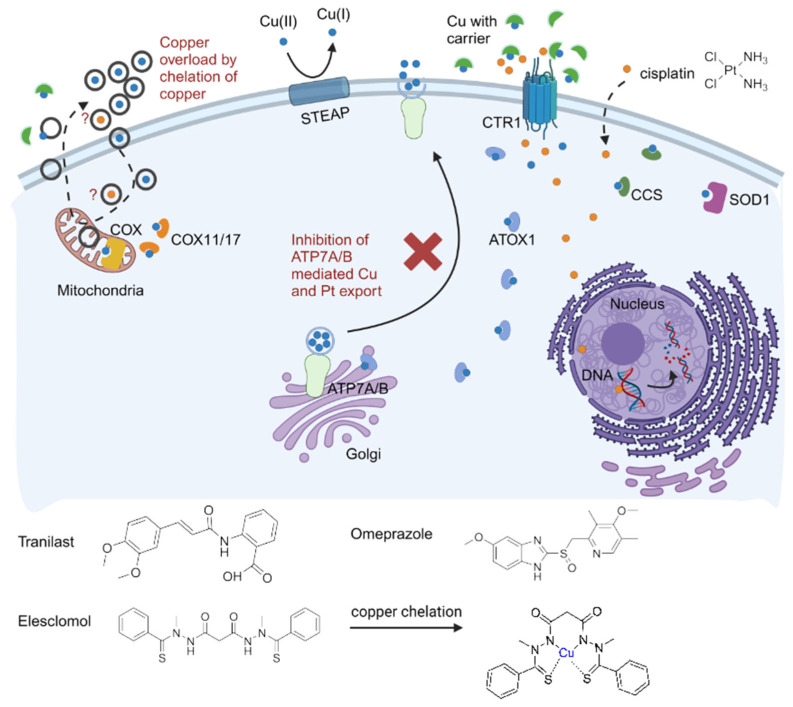
The following simplified sketch illustrates the uptake and transport of copper and cisplatin in the cell, as well as our hypothesis. Cu(II) is reduced to Cu(I) by metalloreductase STEAP. Copper and cisplatin are both transported into cells by the CTR1 transporter, with cisplatin also entering via passive diffusion. Once inside the cell, copper is bound by ATOX1 and can be exported by ATP7A/B. CCS and COX11/17 are responsible for transporting copper to specific targets, namely SOD1 and COX. Cisplatin exerts its cytotoxic effects by binding to DNA. Strategies to enhance cisplatin’s effectiveness include the use of tranilast or omeprazole to inhibit copper export via ATP7A/B, or the employment of the chelator elesclomol, which binds copper and moves it across the cell membrane. Created in BioRender. Doctor, A. (2024). https://BioRender.com/u25i820, accessed on 9 October 2024.

Based on these findings, this preclinical study aimed at investigating the impact of these compounds on the copper transport mechanism of PDAC models (Figure 1). The objective included two approaches. First, two potential ATP7A or B inhibitors, TR and OM, were employed, as ATP7A is responsible for copper transport from the trans-Golgi network to the plasma membrane, where it pumps out excess copper. Consequently, by inhibiting ATP7A, the efflux of copper is blocked. Second, the copper chelator ES was used to elevate intracellular copper levels. The chelator can bind copper and move through the cell membrane, shuffling back and forth [45]. In order to reproduce the heterogeneous and stroma-rich nature of PDAC, the PDAC cell line PanC-1 and the cancer-associated pancreatic stellate cell line HPaSteC, as well as a co-culture of both, were employed in this study [46,47]. Further, HepG2, a hepatocellular cancer cell line that expresses ATP7B [22], served as a reference model [48]. The dynamics of cellular copper uptake and efflux in vitro were monitored using a [^64^Cu]CuCl_2_ radiotracer approach. These combined approaches were applied to test the hypothesis that copper transport mechanisms may represent another druggable target for combination therapies for PDAC.

## 2. Materials and Methods

### 2.1. Cell Culture

The pancreatic ductal cancer cell line PanC-1 was purchased from ATCC (CRL-1469, Manassas, VA, USA). Pancreatic stellate cells HPaSteC were purchased from ScienCell (#3830, Carlsbad, CA, USA). BxPC3 (CRL-1687) and HepG2 (HB-8065) were purchased from ATCC. PanC-1 cells were cultured in Dulbecco’s modified Eagle‘s medium (DMEM), BxPC3 cells in Roswell Park Memorial Institute (RPMI) 1640 medium, and HepG2 cells in Eagle’s minimum essential medium (EMEM). All three types of media were supplemented with 10% fetal calf serum (FCS) (Sigma Aldrich F7524, St. Louis, MO, USA) and 1% penicillin/streptomycin (10,000 U/mL, 10 mg/mL) solution (Biochrom, Berlin, Germany). HPaSteC cells were cultured in SteCM media supplemented with 2% of fetal bovine serum (ScienCell, Cat. No. 0010), 1% of stellate cell growth supplement (SteCGS, ScienCell, Cat. No. 5352), and 1% of antibiotic solution (ScienCell, Cat. No. 0503). Cell lines were routinely analyzed by PCR for mycoplasma contamination. Cell incubation was conducted in a cell incubator under standard cell culture conditions (37 °C, 5% CO_2_, and 95% humidity).

### 2.2. Reagents

The drug compounds tranilast (Sigma-Aldrich, T0318), omeprazole (Selleck Chemicals LLC, S1389, Houston, TX, USA), and elesclomol (MedChemExpress LLC, HY-12040, Monmouth Junction, NJ, USA) were used without further purification and dissolved in sterile DMSO as 50 µg/mL stock solutions. From these stock solutions, the working concentrations for a final concentration of 1% (*v*/*v*) DMSO in cell medium were established. The concentration ranges used in this study were chosen according to the published effective range. For TR, five concentrations from 0 to 305.5 µM were used [49,50]. For OM, concentrations ranging from 0 to 347 µM were employed [43,51], while for ES, concentrations spanning from 0 to 40 nM were utilized [44,52]. Cisplatin (Sigma-Aldrich, C2210000) was dissolved in sterile 0.9% saline at a concentration of 75 µg/mL (249.95 µM). This stock solution was then further diluted.

### 2.3. Copper-64 Accumulation In Vitro

Cells were seeded in 24-well microplates (HPaSteC 100,000 cells/mL, PanC-1 40,000 cells/mL, co-culture 1:3 100,000 cells/mL). After two days and a visual confluency of 80–90%, the medium was removed and the cells were washed with PBS. Then, medium containing either TR, OM, or ES was added and incubated on the cells for 30 min. Subsequently, the medium was removed, and medium containing [^64^Cu]CuCl_2_ (0.05 MBq/well) was added. After a four-hour incubation at 37 °C, the medium was removed and the cells were either washed three times with ice-cold PBS containing 0.9 M CaCl_2_ and 0.5 M MgCl_2_ and lysed with 0.1 M NaOH/1% SDS on ice, or fresh medium was added to monitor the efflux of [^64^Cu]CuCl_2_. For efflux measurements, the medium was removed and the cell lysates were analyzed in the gamma counter after 20 min. The decay-corrected ^64^Cu activity in the cell lysates was quantified using the gamma counter Wizard (PerkinElmer, Waltham, MA, USA). The protein levels were quantified using the BCA assay (Thermo Fisher Scientific, Waltham, MA, USA). The cellular uptake was stated as the percentage of applied dose per mg of protein.

### 2.4. Western Blot Analyses

ATP7A, ATP7B, CTR1, ATOX1, collagen I, and fibronectin were detected by SDS-PAGE followed by Western blotting as reported elsewhere [53]. The membrane was probed with the primary antibody for ATP7A (PA5-36558, Thermo Fisher Scientific, Waltham, MA, USA), ATP7B (ab124973, Abcam, Cambridge, UK), CTR1 (NB100-402, novusbio, Minneapolis, MN, USA), ATOX1 (ab154179, Abcam), collagen I (ab138492, Abcam), fibronectin (ab2413, Abcam), and anti-β-actin (ab5316, Abcam) and with the corresponding secondary antibody conjugated to horseradish peroxidase. Finally, the proteins were visualized by chemiluminescence using SuperSignal^®^ West Pico and Femto Chemiluminescent Substrate (Thermo Fisher Scientific) and imaged using an MF-ChemiBIS Bio-Imaging System (Biostep GmbH, Jahnsdorf, Germany). Western blot images were evaluated using densitometric analysis, performed via GelAnalyzer 23.1.1 with a baseline substation. The resulting intensity values were used to calculate the densitometric index for each protein of interest by division with the values for β-actin.

### 2.5. Cell Viability Assay

The CellTiter-Glo^®^ 2.0 cell viability assay was used according to the manufacturer’s protocol [54]. After monolayer cells (starting density 10,000 cells/mL) were incubated for 72 h at 37 °C in a 96-well plate with the drug (OM, TR, or ES) and cisplatin, CellTiter Glo^®^ 2.0 reagent (Promega, Madison, WI, USA) was added in an amount equal to the volume of the cell culture medium. The solution was mixed to induce cell lysis and incubated for 10 min at 37 °C. The cytation 5 plate reader (Agilent BioTek, Santa Clara, CA, USA) was then used to measure luminescence. Viability was calculated as the difference in luminescence between treated and untreated (reference) cells. Experiments were performed in quadruples.

### 2.6. Cell Confluency

Cells were incubated in 96-well plates for 72 h with a combination of 10, 20, 30, 40, and 50 µM cisplatin, respectively, and 1500 µM TR, 347 µM OM, or 40 nM ES. Moreover, cells with just cisplatin or the drug or the vehicle control (DMSO) were incubated. The confluency was measured at intervals of 6 h and analyzed using an IncuCyte (Sartorius AG, Göttingen, Germany). Experiments were performed in quadruples.

### 2.7. Acidic Activation of OM

The conversion of prodrug OM into the active form sulfenic acid/sulfenamide was conducted according to a protocol published by Lindberg et al. [55]. In brief, OM was dissolved in methanol (145 mM) and then mixed with 4 mM hydrochloric acid and kept at 37 °C for 15 min. Samples were analyzed using UPLC/MS.

### 2.8. UPLC/MS Analyses

The identification of OM and its metabolites was conducted using UPLC with subsequent MS analysis. The UPLC I-Class (Waters Corporation, Milford, MA, USA) was employed, comprising a binary gradient pump BSM, autosampler FTN, column manager CM, and diode array detector PDAeλ. These were coupled to the Waters Xevo TQ-S, with a flow rate of 0.4 mL/min at 50°C and an Aquity UPLC^®^ BEH C18 column (Waters Corporation, Milford, MA, USA, 100 mm × 2.1 mm, 1.7 µm, 130 Å). The eluents were (A) water and (B) acetonitrile. The gradient (%B/%A) was as follows: t_0min_ 25/75–t_0.5min_ 25/75–t_5.5min_ 75/25–t_6.0min_ 95/5–t_7.0min_ 95/5–t_8.0min_ 25/75–t_8.5min_ 25/75. ESI+ mode was used throughout. MassLynx (v4.2 SCN986, Waters) was used for data processing. The samples were diluted to a concentration corresponding to 880 µM non-degraded OM in 45% MeCN/H_2_O (*v*/*v*).

### 2.9. HPLC Analyses

Analytical HPLC was conducted on the Shimadzu Prominence modular HPLC system (Shimadzu Corporation, Kyoto, Japan): degasser DGU-20A5R, 2× pump LC-20AR, autosampler SIL-20AC HT, column oven CTO-20AC with column switching valve, diode array detector SPD-M20A, fluorescence detector RF-20A, and fraction collector FRC-10A, communication bus module CBM-20A, column C-18 Jupiter Proteo (Phenomenex Inc., Torrance, CA, USA; 250 mm × 4.6 mm, 4 µm, 90 Å), flow rate = 1 mL/min, and gradient 0.1% TFA in acetonitrile/0.1% TFA in water: t_0min_ 25/75–t_5min_ 25/75–t_30min_ 95/5–t_34min_ 95/5–t_37min_ 25/75–t_42min_ 25/75. LabSolutions Software (V. 5.92, Shimadzu) was used for data processing.

### 2.10. Statistical Analysis

Statistical analysis was conducted using Prism 10.1.2 (GraphPad Software, San Diego, CA, USA). Differences were tested for significance using an ANOVA and Tukey post-hoc test or ANOVA and Sidak’s test for multiple comparisons or *t*-test. Significance was considered at *p*-values < 0.05.

## 3. Results

### 3.1. Expression of Copper Transport Proteins in PanC-1, HPaSteC, and HepG2

**Figure 2:**
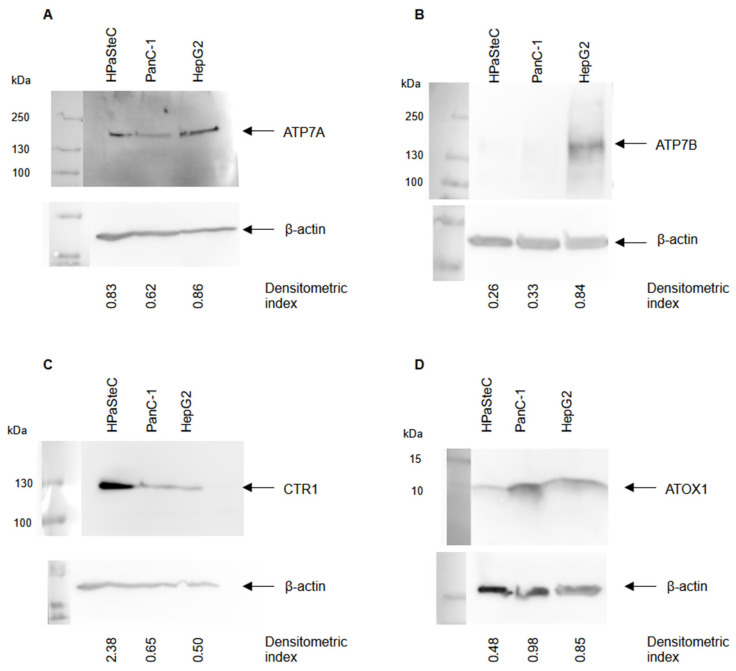
Representative Western blot images of HPaSteC, PanC-1, and HepG2 cells. The membranes were probed with antibodies against copper transport proteins ATP7A (**A**), ATP7B (**B**), CTR1 (**C**), and ATOX1 (**D**). The blots were also probed with an antibody against ß-actin as a control for equal loading. The blots were analyzed via densitometry, and the data were related to β-actin as the loading control. *n* ≥ 4, mean densitometric index.

SDS-PAGE and subsequent Western blot analyses with antibodies against copper transport proteins ATP7A, ATP7B, CTR1, and ATOX1 (Figure 2) demonstrate a more pronounced expression of ATP7A in HPaSteC and HepG2 cells relative to PanC-1. ATP7B, which is predominantly expressed in the liver, was expressed in HepG2 cells only. Of note, CTR1 was expressed in all three cell lines, with HPaSteC cells exhibiting a high expression of CTR1. ATOX1 was found in all three cell lines with a similar level of expression.

### 3.2. Modulation of Intracellular Copper Levels by Inhibition of ATP7A/B Expression

**Figure 3:**
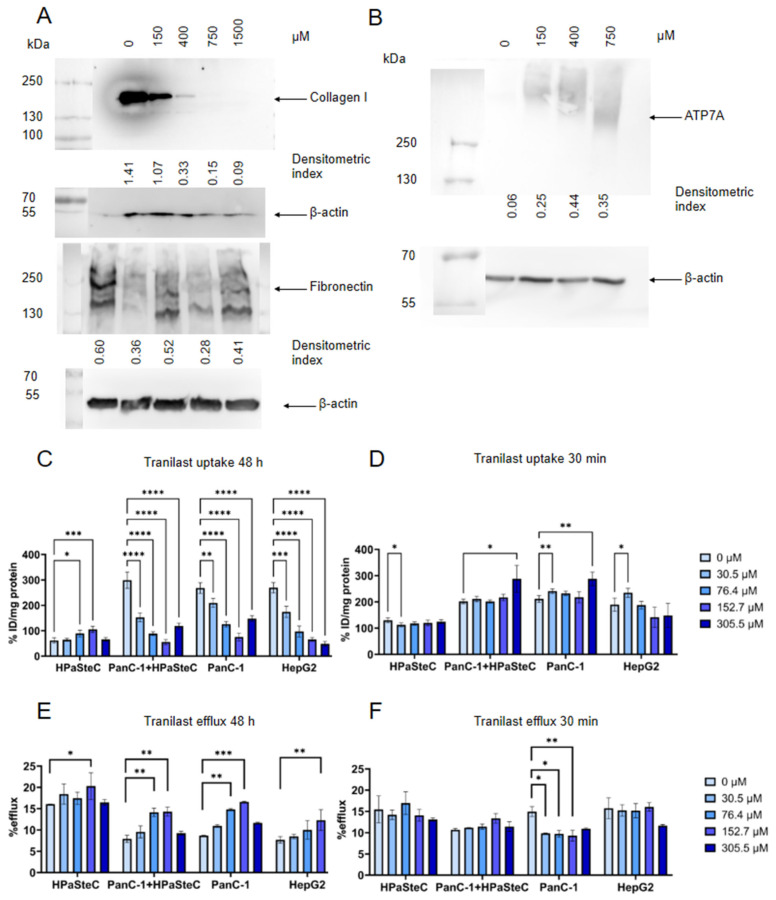
In vitro incubation of PanC-1, HPaSteC, PanC-1+HPaSteC, and HepG2 with TR. (**A**) Western blot of expression of collagen I and fibronectin in HPaSteC cells after 48 h incubation with TR. (**B**) Western blot of expression of ATP7A in PanC-1 cells after 48 h incubation with TR. Analysis of the blots was performed via densitometry with β-actin as loading control (*n* = 4, mean densitometric index). (**C**) [^64^Cu]CuCl_2_ uptake after 48 h incubation with TR. (**D**) [^64^Cu]CuCl_2_ uptake after 30 min incubation with TR. (**E**) [^64^Cu]CuCl_2_ efflux 20 min after 48 h incubation and (**F**) 30 min incubation with TR in a concentration range from 0 to 305 µM. Statistical significance is stated between a concentration of 0 µM and the respective concentrations (*n* = 6, *p* < 0.05, two-way ANOVA, *p*-values are indicated as follows: **** < 0.0001, *** < 0.001, ** < 0.01, and * < 0.05).

TR is reported to be an anti-fibrotic drug that inhibits collagen I and fibronectin expression in cells [56]. PSCs play an important role in the desmoplastic reaction of PDAC. To investigate the possible effect of TR on HPaSteC, cells were incubated with TR at different concentrations for 48 h. The corresponding Western blots are shown in Figure 3A. The expression of collagen I is significantly reduced at higher concentrations and completely inhibited at 250 µg/mL (763 µM). Fibronectin is also reduced in a concentration-dependent manner and is completely inhibited at 500 µg/mL (1.5 mM). It is noteworthy that this long incubation time also has an effect on ATP7A (Figure 3B). A concentration-dependent increase in ATP7A expression was observed. This correlated well with the significant and concentration-dependent decrease in [^64^Cu]CuCl_2_ uptake observed in PanC-1, co-culture, and HepG2 cells and a correspondingly increased copper efflux in PanC-1 (Figure 3C,E). This contradicts our original assumption. Consequently, a shorter incubation time of 30 min was chosen for the subsequent experiments. With this reduced incubation time, a slight but significant increase in [^64^Cu]CuCl_2_ uptake for PanC-1, along with a decrease in efflux, was observed (Figure 3D,F).

**Figure 4:**
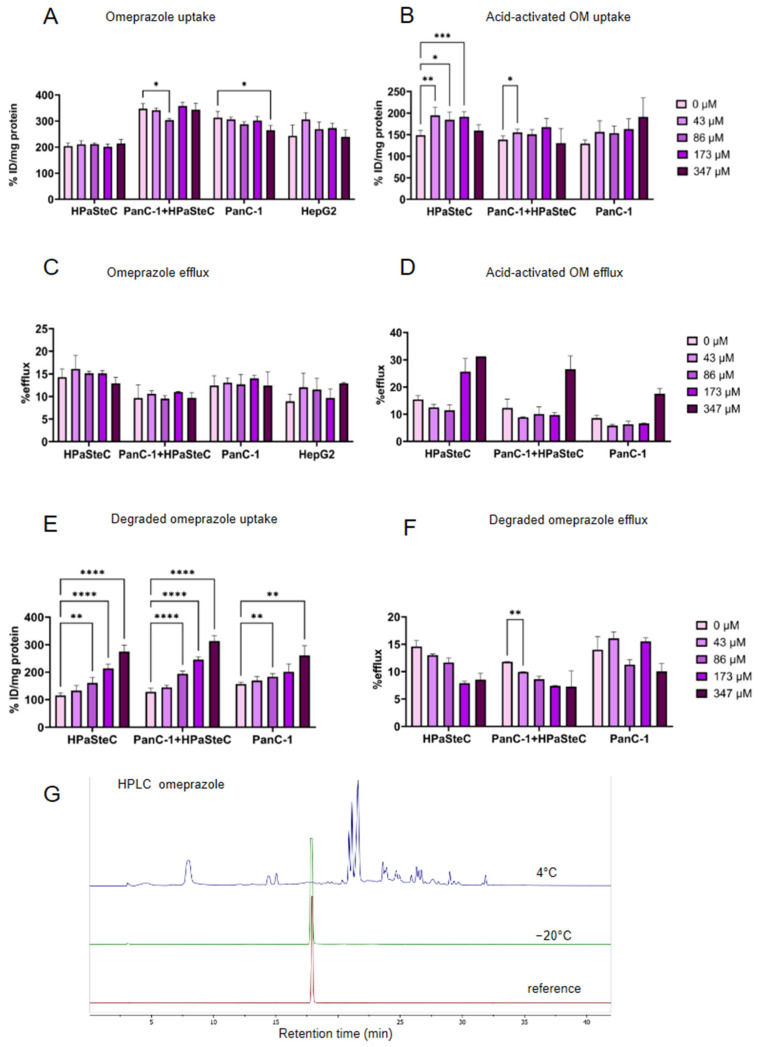
In vitro incubation of PanC-1, HPaSteC, co-culture, and HepG2 with OM. (**A**) [^64^Cu]CuCl_2_ uptake after 30 min incubation with OM. (**B**) [^64^Cu]CuCl_2_ uptake after 30 min incubation with acid-activated OM. (**C**) [^64^Cu]CuCl_2_ efflux 20 min after 30 min incubation with 0 to 347 µM OM. (**D**) [^64^Cu]CuCl_2_ efflux 20 min after 30 min incubation with 0 to 347 µM acid-activated OM. (**E**) [^64^Cu]CuCl_2_ uptake after 30 min incubation with disintegrated OM stored at 4 °C. (**F**) [^64^Cu]CuCl_2_ efflux 20 min after 30 min incubation with 0 to 347 µM disintegrated OM. Statistical differences are stated between a concentration of 0 µM and the respective concentrations (*n* = 6, *p* < 0.05, two-way ANOVA, *p*-values are indicated as follows: **** < 0.0001, *** < 0.001, ** < 0.01, and * < 0.05). (**G**) HPLC of freshly dissolved OM (reference), disintegrated OM stored at 4 °C, and OM stored at −20 °C.

As TR treatment resulted in only a slight increase in [^64^Cu]CuCl_2_ uptake even at high concentrations, a second potential ATP7A inhibitor, OM, was tested. The original prodrug OM, however, had no accumulative or efflux reducing effect on [^64^Cu]CuCl_2_ uptake by the cells in the present experimental setting (Figure 4A,C). The activation process of OM as a proton pump inhibitor (PPI) is acid-catalyzed and leads to the formation of a sulfenamide which is discussed to be the active species in the inhibition of ATPases. In an attempt to reproduce the activation process, the acid-sensitive OM was incubated over 15 min at 37 °C with 4 mM HCl leading to its complete decomposition. Tested on cells, a significant increase in [^64^Cu]CuCl_2_ was observed for HPaSteC cells at concentrations between 43 and 173 µM (Figure 4B), but no efflux-reducing effect (Figure 4D). An OM stock solution in DMSO was found to be stable at −20 °C as shown by HPLC and comparison to a freshly prepared stock solution (Figure 4G). When stored at 4 °C, the solution contained various degradation products and only trace amounts of the intact OM (Figure S1). Also, a change in color could be observed from translucent to yellow brownish. UPLC/MS analysis of the degraded OM (dOM) showed m/z signals corresponding to various degradation products of the intermediately formed sulfenic acid and its dimerization products as they were described by Brändström et al. [57]. UPLC/MS analysis of the acid-activated OM sample showed the degradation of OM into five UV-detectable metabolites, four of which were also found in dOM, among a variety of others (Figures S1–S3). Interestingly, however, this degraded compound (dOM) showed an ability to enhance copper accumulation in cells (Figure 4E) and showed a trend to reduce the tracer efflux (Figure 4F). Incubation with dOM resulted in increased [^64^Cu]CuCl_2_ uptake with concentration above 43 µM for HPaSteC, co-culture, and PanC-1 cells and, moreover, a decreased efflux with increasing dOM concentration. Due to the complexity of the degradation processes described therein, as well as the number of species found, the exact nature of the degradation products was not further elucidated.

### 3.3. Modulation of Intracellular Copper Levels by Copper Chelation

**Figure 5:**
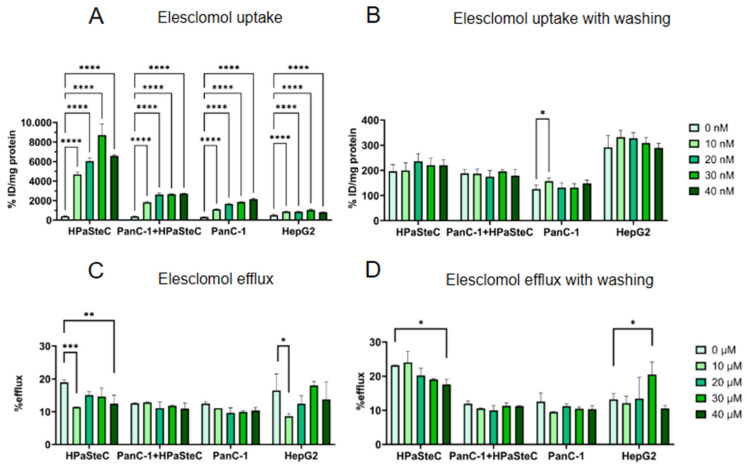
In vitro incubation of PanC-1, HPaSteC, co-culture, and HepG2 with ES. (**A**) [^64^Cu]CuCl_2_ uptake after 30 min incubation with ES. (**B**) [^64^Cu]CuCl_2_ uptake after 30 min incubation with ES followed by removal of ES through washing. (**C**) [^64^Cu]CuCl_2_ efflux at 20 min after 30 min incubation with ES. (**D**) [^64^Cu]CuCl_2_ efflux at 20 min after 30 min incubation with ES followed by removal of ES. Statistical differences are stated between a concentration of 0 µM and the respective concentrations (*n* = 6, *p* < 0.05, two-way ANOVA, *p*-values are indicated as follows: **** < 0.0001, *** < 0.001, ** < 0.01, and * < 0.05).

In a complementary approach to enhance copper uptake, the influence of ES was investigated, which acts as a chelator or ionophore and supports shuffling copper into the cells. As shown in Figure 5A, a significant increase in [^64^Cu]CuCl_2_ uptake was achieved in the nM concentration range for all four cell types, in particular for HPaSteC (400 %ID/mg protein to 8700 %ID/mg protein at a concentration of 30 nM). Copper efflux was significantly reduced in HPaSteC and HepG2 cells (Figure 5C). Based on a publication showing that ES degrades ATP7A [44], it was tested whether this was the reason for the significant accumulation of [^64^Cu]CuCl_2_. ES was removed by washing prior to incubation with [^64^Cu]CuCl_2_ (Figure 5B,D). This results in a significant copper uptake in PanC-1 at 10 nM and a significantly reduced copper efflux in HPaSteC cells incubated with 40 nM ES, but an increased copper efflux in HepG2 cells. No other effects were found.

In order to determine whether the modulation of copper accumulation by ES observed in vitro could be replicated in vivo, a pilot experiment was conducted on tumor-bearing mice. [^64^Cu]CuCl_2_ accumulation, with and without ES, was measured using small animal positron emission tomography. The results and discussion are presented in the Supplementary Materials (Figure S4).

### 3.4. Cellular Anticancer Acitivity of Cisplatin with and Without Copper-Transport Modulating Drugs

**Figure 6:**
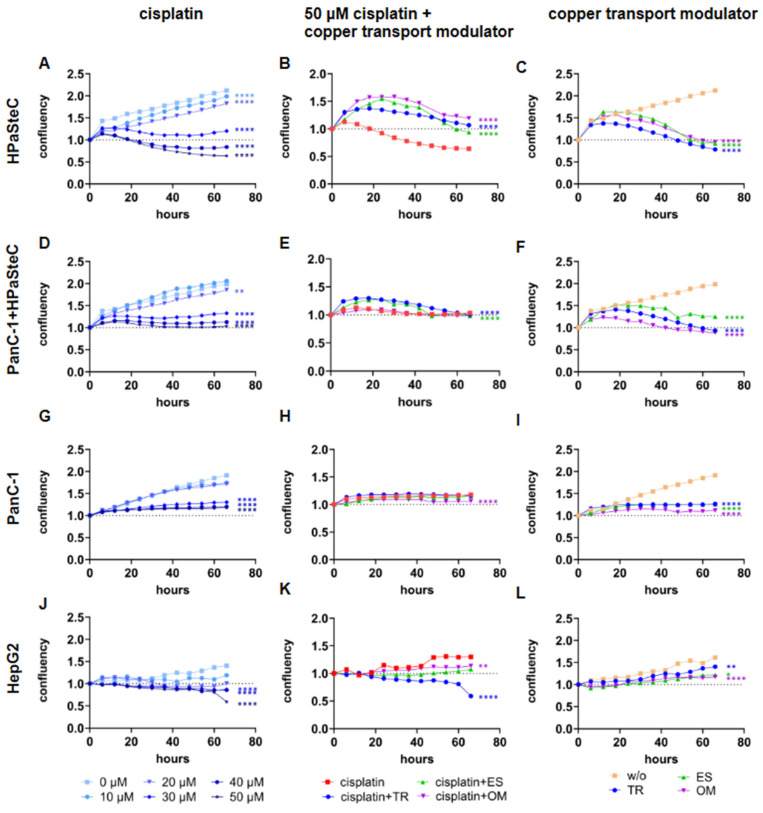
Effect of copper transport modulators and cisplatin on cells. Confluency of HPaSteC (**A**–**C**), PanC-1+HPaSteC (**D**–**F**), PanC-1 (**G**–**I**), and HepG2 (**J**–**L**) cell cultures incubated with cisplatin at concentrations of 0–50 µM for 66 h (**A**,**D**,**G**,**J**). Further, confluency of cell cultures incubated with 50 µM cisplatin and 1500 µM TR, 40 nM ES, or 347 µM OM for 66 h (**B**,**E**,**H**,**K**) and confluency of cell cultures incubated with 1500 µM TR, 40 nM ES, or 347 µM OM for 66 h (**C**,**F**,**I**,**L**). Statistical significance is stated between the AUC of concentration 0 µM and the respective concentrations or conditions (*n* = 4, two-way ANOVA, *p*-values are indicated as follows: **** < 0.0001, ** < 0.01, and * < 0.05).

**Figure 7:**
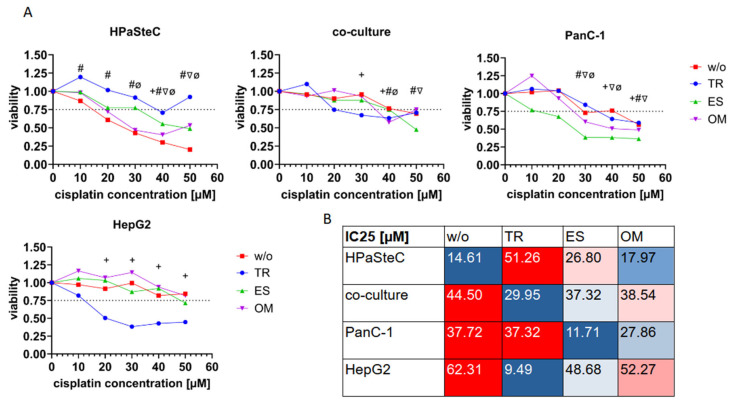
(**A**) Dose–response curves of HPaSteC, co-culture, PanC-1, and HepG2 cells following treatment with cisplatin alone or in combination with 1500 µM TR, 40 nM ES, or 347 µM OM. Statistical significance is stated between a concentration of 0 µM and the respective concentrations (*n* = 4, *p* < 0.05, two-way ANOVA) # *w/o*, + TR, ∇ ES, and ø OM. (**B**) Corresponding IC_25_ values calculated for each treatment condition. An increase in red coloring indicates a high IC_25_, while an increase in blue coloring indicates a low IC_25_.

Given that copper and platinum utilize the same cellular uptake pathways via CTR1 and the same efflux pathways via ATP7A and ATP7B, we postulated that it should be possible to accumulate not only [^64^Cu]CuCl_2_, but also platinum in the form of cisplatin in cells. Furthermore, we hypothesized that this enhanced accumulation of the chemotherapeutic agent would be detectable by an enhanced tumor cell death. To determine this effect, cell confluency and cell viability were analyzed. At this step, the PDAC cell line BxPC3 was included in the experimental setup as a second reference cell line due to its known sensitivity to cisplatin [58]. The results are included in the Supplementary Materials (Figure S5). Figure 6 shows the effect of cisplatin and the three copper modulators on the cell confluency of HPaSteC, co-culture, PanC-1, and HepG2. An effect of cisplatin is observed at concentrations higher than 30 µM. A decrease in confluence is observed for HPaSteC and HepG2 cultures at 40 and 50 µM cisplatin, while a stagnation in confluence is observed for co-culture and PanC-1 (Figure 6A,D,G,J). Further, cell cultures were inoculated with 50 µM of cisplatin and TR, ES, or OM (Figure 6B,E,H,K). A decrease in confluency was observed for the HPaSteC culture incubated with cisplatin, but it did not begin until approximately 24 h of incubation, in contrast to the observation without drugs which showed a decrease after 6 h of incubation. For co-culture confluency, no effect was observed for OM, but TR and ES also prolonged the confluency-decreasing effect of cisplatin to 18 and 24 h, respectively. For PanC-1, a decrease in confluency was observed with OM, while the other two drugs showed no effect. For HepG2, all three drugs decreased the cell confluency more than cisplatin alone. In particular, TR reduced cell confluency from the outset of the incubation period to 40% less confluency after 66 h. Subsequently, the effect of TR, ES, and OM alone on cell confluency was analyzed (Figure 6C,F,I,L). For all cell cultures, we observed an effect on confluence between 12–18 h after incubation. While untreated cells thrived, the growth of HPaSteC and co-culture cells decreased when incubated with the drugs. Stagnation was observed for PanC-1. HepG2 seemed to be the least affected, still showing increasing cell confluency. In order to ensure the reliability of the results, the viability of the cells was assessed in addition to confluency, as it is not always possible to distinguish between dead but still attached cells and viable cells. Figure 7 presents the results of the cell viability test, which was conducted at the endpoint, after 66 h. The change in cell viability is displayed in relation to the concentration of cisplatin. The viability of HPaSteC cells exhibited a continuous and significant decline with increasing cisplatin concentration. In accordance with the degree of cell confluency, the cytotoxic effect of cisplatin was reduced when incubated with TR, OM, or ES. In particular, TR exhibited protective abilities. In co-culture, TR demonstrated lower cell viability for cisplatin concentrations of 30 µM, while ES demonstrated lower cell viability for 50 µM. For the PanC-1 cell line, the cytotoxic effect of cisplatin was enhanced by ES. In accordance with confluency measurements, HepG2 cells exhibited a significant decline in viability when incubated with cisplatin and TR in combination.

Cisplatin dose–response curves were plotted in the absence or in the presence of 1500 µM TR, 40 nM ES, or 347 µM OM (see Figure 7A). The recorded luminescence of the CellTiterGlo assay 66 h after cisplatin incubation was normalized to the control group, which received no additional cisplatin treatment. The fitted IC_25_ values are presented in tabular form in Figure 7B, which shows the IC_25_ values for HPaSteC, co-culture, PanC-1, and HepG2 following treatment with cisplatin alone (*w/o*) and in combination with the drugs TR, ES, and OM. In the case of HPaSteC cells, the untreated control (*w/o*) exhibits an IC_25_ of 14.61 µM. Treatment with TR resulted in a notable increase in the IC_25_, reaching 51.26 µM. This observation suggests a potential reduction in the sensitivity or the presence of a protective effect exerted by TR. Similarly, ES elevates the IC_25_ to 26.80 µM, though to a lesser degree than TR. The IC_25_ for OM treatment is 17.97 µM, which is closer to the control but still exhibits a slight increase. In the co-culture model, the IC_25_ value for the untreated control is 44.50 µM. Treatment with TR resulted in a reduction of the IC_25_ to 29.95 µM, indicating a heightened sensitivity in comparison to the control. Both ES and OM demonstrate comparable IC_25_ values, with 37.32 µM and 38.54 µM, respectively, exhibiting a slight reduction compared to the untreated value. In the case of PanC-1 cells, the IC_25_ value in the absence of additional copper modulator treatment is 37.72 µM. Both TR and OM yielded comparable IC_25_ values, with 37.32 µM and 27.86 µM, respectively. However, the IC_25_ for ES is considerably lower, at 11.71 µM, indicating a heightened sensitivity. The highest IC_25_ values were observed in the untreated control for HepG2 cells (62.31 µM). TR markedly diminishes this value to 9.49 µM, thereby indicating robust sensitivity. Furthermore, ES reduces the IC_25_ to 48.68 µM, whereas OM remains closer to the control at 52.27 µM.

## 4. Discussion

The objective of this study was to investigate cellular copper transport pathways as potential therapeutic targets in PDAC. Therefore, in vitro cell experiments were conducted. The copper transport system was modulated using three different substances, and the dynamics of copper transport were analyzed using the radiotracer [^64^Cu]CuCl_2._ In addition, experiments were performed to investigate the effect of cisplatin on the cell models used, given that cisplatin utilizes the same transport processes as copper. By exploring these interactions, the study aimed to uncover novel strategies to improve therapeutic outcomes for PDAC. The ability of three drugs, TR, OM, and ES, to enhance copper accumulation in PDAC cells, PSCs, and a co-culture of the two as well as ATP7B-expressing liver cancer cell line HepG2 was examined in vitro. For the purpose of examining the effect of copper modulators on the anticancer activity of cisplatin, the PDAC cell line BxPC3, known to be sensitive to cisplatin, was included in the studies at a later step and the results are presented in the Supplementary Materials (Figure S5).

TR ((*E*)-2-[3-(3,4-dimethoxyphenyl)acrylamido]benzoic acid) is used for the treatment of bronchial asthma and for inflammation and hypertrophic scars. TR can inhibit the production of prostaglandins as well as cytokines and chemokines [56]. The treatment of scars with TR suppresses the production of collagen I and III by fibroblasts [59]. As PDAC is a cancer with a high stroma content, the anti-fibrotic properties combined with the inhibited platinum export and low toxicity (600 mg/day over several months is non-toxic to humans [56]) were especially interesting for us. In our study, TR exhibited antifibrotic properties, as evidenced by a concentration-dependent reduction in collagen I and fibronectin expression after 48 h of incubation (Figure 3). Due to its ability to inhibit TGF-β pathways and induce the formation of tumor suppressors p53 and p21, TR has also been tested as an anticancer drug in lung carcinoma [60], osteosarcoma [50], and uterine leiomyoma [61] as well as pancreatic cancer [62]. In ovarian cancer cells, TR inhibited the cisplatin export with ATP7B from the Golgi, without inhibiting the copper export in hepatic cells. Moreover, TR-treated cells showed a downregulation of ATOX1 [42]. In our study, enhanced copper accumulation and reduced efflux were observed for PanC-1 and co-culture, although high concentrations of TR (305 µM) were required.

The second drug that was used for copper uptake modulation is OM (5-methoxy-2-[(4-methoxy-3,5-dimethylpyridin-2-yl)methylsulfinyl]-1H-benzo[d]imidazole). OM was developed in 1982 by Lindberg et al. as the first-in-class of benzimidazole-based PPIs. Nowadays, PPIs are indispensable in the treatment of non-erosive and ulcerating gastroesophageal reflux disorders. OM irreversibly binds to the gastric P-type H^+^/K^+^-ATPase ATP4A, thus inhibiting gastric acid secretion by parietal cells. In neutral pH, OM is an inactive, stable prodrug. However, in acidic conditions, it undergoes a series of cyclization and ring-opening reactions forming a sulfenic acid which is in equilibrium with an intramolecular sulfenamide. According to Lindberg et al., both of these species can bind to ATP4A via cysteine residues, though more recent studies suggest that only the sulfenic acid is active and its transition to the sulfenamide is irreversible [63,64,65]. It has been published that OM blocks ATP7A-dependent melanogenesis in melanoma cells as well as ATP7A trafficking [43]. Moreover, the treatment of mice bearing melanoma xenograft tumors with OM increased sensitivity towards chemotherapy [66].

Matsui et al. describe that OM is capable of inhibiting melanogenesis in melanocytes, which they attributed to APT7A inhibition and the subsequent reduction in the metalation of tyrosinase [19]. The effects of decreased tyrosinase synthesis were only observed following an exposure to OM of 48–72 h. The authors attributed this to the turnover of existing tyrosinase and accumulation, as well as to the molecular activation processes of OM [43]. This conclusion is consistent with our findings. While the intact PPI demonstrated no evidence of elevated copper retention within the cells, the dOM solution exhibited an increase in copper retention. It is noteworthy that the mixture of acid-activated OM metabolites did not elicit a comparable effect on the cells, indicating that other metabolites than those formed during acid degradation are responsible for the observed effect (Figure 4). This can be explained by the fact that the sulfenic acid and sulfenamide are permanent cations that are unable to penetrate the cell membrane [55]. It can be reasonably concluded that the active compound in the degraded OM sample is most likely an uncharged metabolite that is capable of overcoming the cell membrane.

Thirdly, the copper chelator ES (1-N′,3-N′-dimethyl-1-N′,3-N′-di(phenylcarbonothioyl)malonohydrazide) was used. ES is a tetradentate copper ionophore that can transport extracellular copper(II) ions [67,68]. The formed 1:1 complex is uncharged and can cross membrane barriers. In the mitochondria, the ES-bound copper is released after reduction to Cu(I). ES then shuffles back into the extracellular environment to bind more copper [45]. ES is investigated as an adjuvant in chemotherapy, mainly in combination with taxanes but also with platinum-based drugs [69]. The precise mechanism by which ES exerts its anticancer effects remains unclear. One potential explanation is that ES acts as a cuproptosis inducer. Cuproptosis is a process whereby lipoylated tricarboxylic acid (TCA) enzymes bind copper, which results in protein aggregation and proteotoxic stress. Cancer cells with a high level of lipoylated TCA enzymes due to mitochondrial respiration exhibit increased sensitivity to cuproptosis [70]. In our study, ES demonstrated remarkable efficacy in enhancing copper accumulation in the nanomolar range. In particular, HPaSteC cells exhibited a more than 21-fold increased copper accumulation at 30 nM, in comparison to the copper uptake capacity of cells without ES (Figure 5). As ES was previously reported to promote the degradation of ATP7A [44], it was tested if this could also be observed. Consequently, ES was removed prior to copper incubation through washing. No substantial difference in copper accumulation was observed, indicating that the elevated accumulation with ES in this experimental setup is attributed to the chelating effect and not to the degradation of ATP7A (Figure 5B).

Subsequently, the impact of the pharmaceutical agents on platinum anti-cancer activity was assessed. Platinum-containing drugs are a common component of cancer therapy regimens. Some tumors including PDAC are resistant to the effects of those drugs. In multiple publications, it has been demonstrated that platinum resistance is linked to copper transport mechanisms. Platinum can be transported by CTR1, and the expression of ATP7A and B as well as ATOX1 is responsible for the treatment response of platinum-containing drugs [36,39,71,72,73,74]. Therefore, an anticancer activity assessment was conducted by incubating the cells with varying concentrations of cisplatin (*cis*–diaminedichloridoplatinum(II)) and the three drugs. Subsequently, the degree of cell confluency and the viability of the cells, as measures for the therapeutic effect, were evaluated. The cells exhibited a reduction in confluency at cisplatin concentrations above 30 µM. HPaSteC cells exhibited the greatest impact, indicating greater sensitivity to cisplatin than the cisplatin-sensitive PDAC cell line BxPC3 (see Supplementary Materials and Figure S5). This finding is particularly intriguing in light of the prevailing view that PSCs are the underlying cause of the chemoresistant features observed in PDAC [75,76]. To date, no data on the impact of cisplatin on PSCs is available. In light of a recent publication by Zisowsky et al. [77] concerning the influence of CTR1 expression on cisplatin sensitivity, the relatively high CTR1 expression observed in HPaSteCs (Figure 2) may provide a potential explanation for their high cisplatin sensitivity. Of note, due to the chemically distinct properties of copper ions and cisplatin, the mechanisms of CTR1-mediated uptake from the extracellular space to the cytoplasm also differ. The homotrimeric ion channel features a copper selectivity filter, extracellular methionine- and histidine-rich copper-binding domains, and intracellular gating domains aiding in copper ion distribution to chaperones such as ATOX1 [78,79,80]. Cisplatin likely interacts with the methionine-rich domains but does not elicit a similar conformational change as copper ions in the intracellular gating domains. Taken together with its size precluding cisplatin from passage through the ion channel, distinct mechanisms such as endocytosis after binding to CTR1, the multimeric reorganization of CTR1, and passage after ligand exchange enabled by the trans effect of methionine sulfur were suggested for CTR1-mediated platinum transport [23,81,82,83,84].

The incubation of cells with TR, OM, or ES resulted in a reduction in confluency and viability (Figure 6 and Figure 7). These results demonstrate that the drugs can exhibit anticancer activity when used alone. It is likely that this effect is attributable to cuproptosis. No additional copper was added during the incubation period, but copper is available through the serum added to the standard commercial cell media [85]. A notable decline in cell confluency was observed when the drugs or cisplatin were administered. The drugs demonstrated an earlier impact of approximately 6 h in contrast to cisplatin which exhibited effects after approximately 24 h. This can be explained by the fact that cuproptosis leads to protein toxic stress and eventually cell death, indicating a relatively quick impact on cell viability once the process is initiated [86,87], whereas studies suggest that cisplatin can significantly impact cell viability within 24 to 48 h [88,89].

The addition of TR, OM, and ES to cisplatin resulted in a notable reduction of cytotoxicity in HPaSteC cells but a significant increase in cancer cells. Further research is needed to explain the reasons behind this. ES was particularly efficacious in additionally increasing the anti-cancer activity of cisplatin. All tested cancer cell lines exhibited a lower IC_25_ for the combination therapy of ES and cisplatin compared to the IC_25_ of cisplatin alone. For instance, PanC-1 cells showed a confluency reduction of 36% and a reduced IC_25_ from 37.7 to 11.7 µM. The co-culture exhibited a complex set of effects, resulting from the opposing effects of HPaSteC and PanC-1 on the treatment. HepG2 showed enhanced anticancer activity with the combination of TR and cisplatin with a reduced IC_25_ from 62.3 to 9.5 µM. This can be explained, as TR has previously been shown to affect ATP7B [42] and HepG2 cells express ATP7B (Figure 2).

These findings illustrate the potential of copper modulators to improve the efficacy of cisplatin, as evidenced by the reduced IC_25_ values. Furthermore, the co-culture results underscore the role of HPaSteC in promoting chemoresistance in PDAC cells, emphasizing the necessity of incorporating co-culture models in preclinical therapeutic studies.

Following this, a pilot experiment was performed in tumor-bearing mice. The aim was to determine whether the in vitro modulation of copper accumulation by ES could be transferred to the in vivo situation. The in vivo [^64^Cu]CuCl_2_ transport with and without ES, which showed the most prominent in vitro results, was measured by small animal positron emission tomography (see Supplementary Materials and Figure S4). However, the data showed a significantly increased [^64^Cu]CuCl_2_ radiotracer accumulation in the brain, but not in the tumor. This result is consistent with a recently published paper by Liu et al. [90]. As previously documented [91], the physiological uptake of copper in the brain is minimal. The elevated levels of ^64^Cu in the brain following ES administration are presumably attributable to an increase in cytochrome c oxidase levels within the brain and the capacity of ES to traverse the blood–brain barrier [92]. Therefore, in vivo experiments with ES in combination with cisplatin were not performed.

## 5. Conclusions

In conclusion, our findings demonstrate that the modulation of copper transport mechanisms with the drugs TR, ES, and OM can enhance both copper uptake and platinum-induced anticancer activity in cells in vitro. Each drug exhibited inherent anticancer activity, likely driven by cuproptosis via three mechanisms: ATP7B inhibition by TR, ATP7A inhibition by OM, and copper overload by the chelating agent ES. Moreover, the combined treatment of these drugs with cisplatin resulted in increased anticancer activity in PDAC cells, while cancer-associated PSCs were comparatively spared, suggesting a protective mechanism in the latter that needs further investigation. Although the use of only selected cell lines cannot fully reflect the unique heterogeneity of this tumor stromal entity, the results suggest that targeting copper transport mechanisms could offer new adjuvant approaches for the treatment of PDAC. The possibility of using combinations of already approved drugs appears particularly attractive.

## 6. Limitations of the Study

The present study naturally has limitations, the most important of which are briefly outlined here. Cell-based studies with cisplatin and other agents provide valuable mechanistic data and can help identify potential synergies. However, preclinical findings with cisplatin cannot always predict the clinical outcome of oxaliplatin-based therapies.

Although both cisplatin and oxaliplatin are platinum-based chemotherapeutic agents, oxaliplatin has a different chemical structure, which alters its pharmacological properties and side effect profile compared to cisplatin. Resistance mechanisms to cisplatin and oxaliplatin may overlap but are not identical. This limits the transferability of cell-based studies with cisplatin in combination with other agents, for example, to the clinical application of the FOLFIRINOX/mFOLFIRINOX treatment regimens containing oxaliplatin instead of cisplatin.

The drug concentrations reported in the manuscript were chosen to cover a broad dosage range. However, since we work in static in vitro models, it is likely that significantly higher concentrations are present in cell culture than under dynamic in vivo conditions.

We are also aware that in vitro conditions are fundamentally not comparable to in vivo conditions. The complexity and heterogeneity of the tumor-stromal entity PDAC cannot be remotely reproduced with the selected cell lines and under the experimental conditions used. This also applies to the pharmacological properties of the compounds investigated, which may also influence each other and cannot be simulated in vitro. Therefore, the results obtained here can only provide an initial assessment.

However, they show clear and significant effects that justify the conclusions discussed above, in particular the connection between the copper transport mechanism and platinum-induced anticancer activity. The extent to which [^64^Cu]CuCl_2_ can contribute as an imaging marker to the in vivo characterization of copper transport has also only been touched upon here. Further studies would need to be conducted in reliable animal models. In our view, however, a conclusive evaluation is only possible in patients.

## Data Availability

The data presented in this study are contained within the article.

## Supplementary Materials

The following supporting information can be downloaded at https://www.mdpi.com/article/10.3390/cells14191489/s1. Figure S1: UPLC analysis of OM reference solution; Figure S2: UPLC analysis of acid-activated OM; Figure S3: Stacked UPLC-DAD chromatograms (190–800 nm) of omeprazole (bottom, red), acid-activated omeprazole (middle, green) and degraded omeprazole (top, blue). Absorption was normalized to the highest peak; Figure S4: In vivo PET imaging in tumor-bearing mice; Figure S5: Effect of copper transport modulators and cisplatin on BxPC3 cells; Figure S6: The entire collagen I, ATP7A and fibronectin Western blot image is presented here; Figure S7: The entire Western blot image with antibodies against ATP7A (A), ATP7B (B), CTR1 (C) and ATOX1 (D) in HPaSteC, PanC-1 and HepG2 cells is presented here. All blots with corresponding β-actin. References [46,91,92,93,94,95,96] are cited in the Supplementary Materials.
