# Supplementary material for: Investigation of the Putative Relationship Between Copper Transport and the Anticancer Activity of Cisplatin in Ductal Pancreatic Adenocarcinoma
Source: Cells. 2025 Sep 24;14(19):1489. doi: 10.3390/cells14191489 (PMC12523427; doi:10.3390/cells14191489)
Supplement: Supplementary file 1 [file cells-14-01489-s001.zip › cells-3709516-supplementary.pdf]

Supplementary Information

Investigation of the putative relationship between copper transport and the anticancer activity of cisplatin in ductal pancreatic adenocarcinoma

Alina Doctor [1,2], Jonas Schädlich [1,2], Sandra Hauser [1] and Jens Pietzsch [1,2,*]

1 Department of Radiopharmaceutical and Chemical Biology, Institute of Radiopharmaceutical Cancer Research, Helmholtz-Zentrum Dresden-Rossendorf, Bautzner Landstrasse 400, 01328 Dresden, Germany; a.doctor@hzdr.de (A.D.); j.schaedlich@hzdr.de (J.S.), s.hauser@hzdr.de (S.H.)
2 Faculty of Chemistry and Food Chemistry, School of Science, Technische Universität Dresden, Bergstrasse 66, 01069 Dresden, Germany
* Correspondence: j.pietzsch@hzdr.de (J.P.)

1. Omeprazole degradation observed via UPLC-MS

Only mass signals that could be assigned to the structure of intact OM or a degradation product of OM are shown.

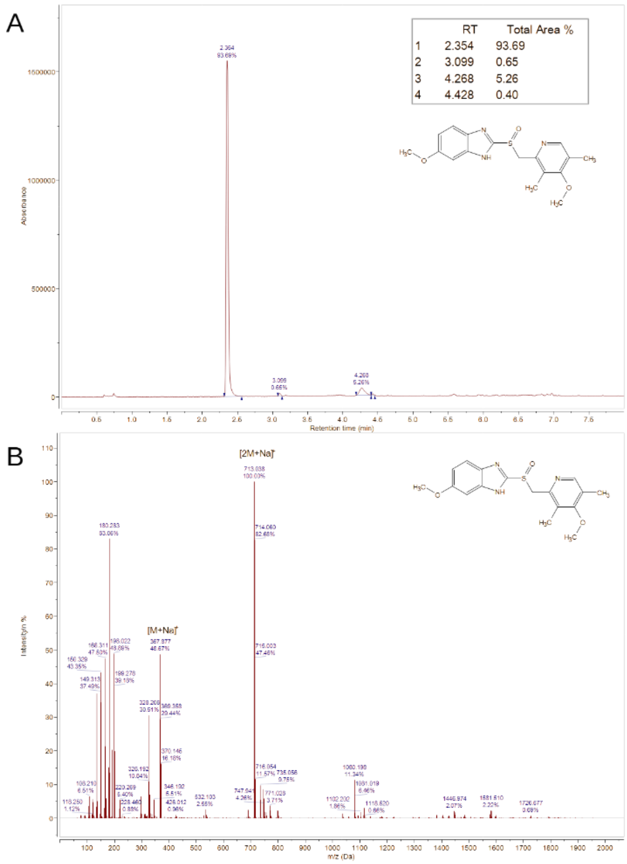

Figure S1: UPLC analysis of OM reference solution. UPLC-DAD chromatogram (254 nm) (A) and mass signal at 2.35–2.40 min extracted from total ion count (TIC) (B) of omeprazole reference solution showing 93.7 % purity (254 nm).

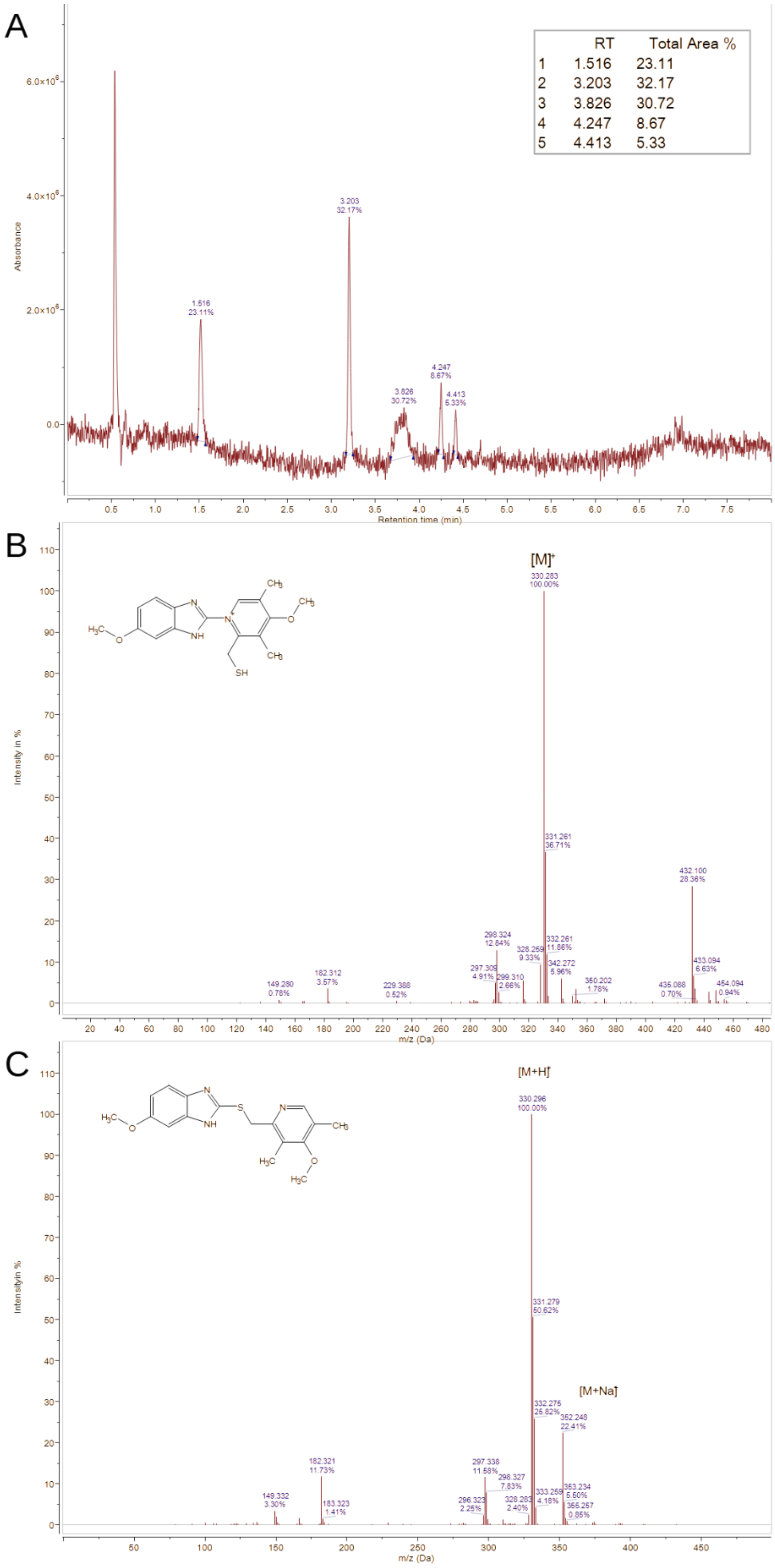

Figure S2: UPLC analysis of acid-activated OM. UPLC-DAD chromatogram (total absorption 190–800 nm) (A) and mass signal extracted from TIC with proposed structure of corresponding degradation product (B+C) at 1.51–1.67 min (B) and 3.16–3.35 min (C) of acid-activated OM (15 min 37°C 4 mM HCl). No intact omeprazole was detected. Mass signals of further peaks could not be attributed to a specific structure.

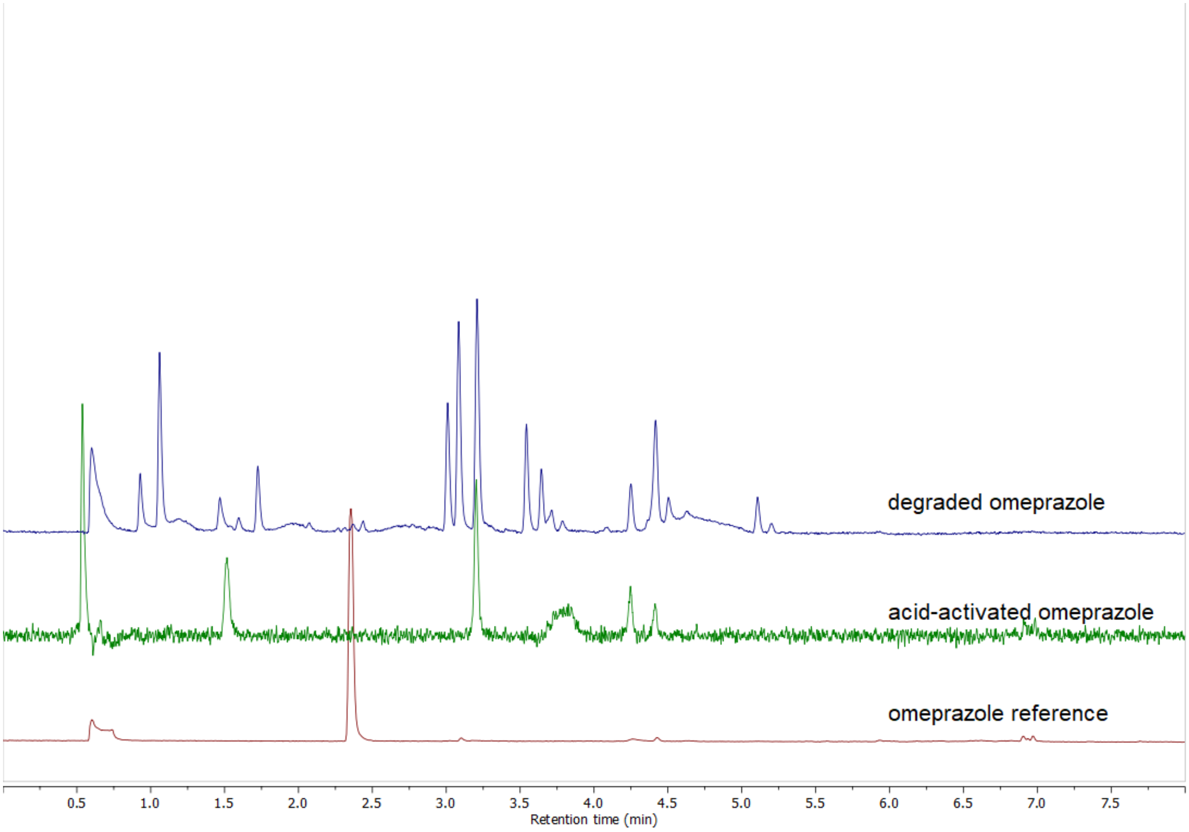

Figure S3: Stacked UPLC-DAD chromatograms (190–800 nm) of omeprazole (bottom, red), acid-activated omeprazole (middle, green) and degraded omeprazole (top, blue). Absorption was normalized to the highest peak.

2. Copper transport modulation with elesclomol in vivo

Following the in vitro studies with copper transport modulators and radiotracer [^{64}Cu]$CuCl_2$, an in vivo pilot experiment was performed in tumor-bearing mice. The aim was to determine whether the in vitro modulation of copper accumulation by ES could be transferred to in vivo experiments.

2.1. Material and Methods

Imaging with PET/CT All animal experiments were carried out according to the German Regulations for Animal Welfare guidelines and have been approved by the local Ethical Committee for Animal Experiments (reference number DD24.1-5131/449/49). The xenograft mouse model was conducted as reported elsewhere [1]. Shortly, female SCID (severe combined immunodeficiency disease) mice were injected subcutaneously with $5*10^6$ cells suspended in 100 µL Dulbecco´s phosphate-buffered saline (PBS). The cell suspension consisted of either PanC-1 cells or a mixture of PanC-1 and HPaSteC cells in a 1:3 ratio. The mice's well-being was monitored daily, and their weight and tumor growth were measured and recorded three times a week. Tumor size was determined by caliper, and tumor volume was calculated using the formula $V = \pi/6 \times abc$, assuming a triaxial ellipsoid with the axes a, b, and c. ES was dissolved in 5% DMSO, 40% PEG300 and 5% Tween 80 and topped with 0.9% NaCl and [^{64}Cu]$CuCl_2$ for a solution containing 50 mg/kg ES. Each animal received an intravenous injection of 250 µl corresponding to 10-12 MBq [^{64}Cu]$CuCl_2$ through a tail vein catheter. Dynamic imaging was performed for 2 hours and after 4h, 24h and 48h. During the imaging procedures, general anesthesia was maintained with inhalation of 9% desflurane in 30/10% oxygen/air. Animals were warmed at 37 °C. Small animal PET was performed using the nanoPET/CT scanner (Mediso Medical Imaging Systems, Budapest, Hungary). With each PET scan, a corresponding computed tomography (CT) image was recorded and used for anatomical referencing and attenuation correction. For binning, framing and image reconstruction, protocols described elsewhere were used [6, 7]. The three-dimensional list-mode data was binned using the 400-600 keV energy window and sorted in up to 36 time frames. The Tera-TomoTM 3D algorithm was used to reconstruct the time frames with corrections for decay, scatter, and attenuation and a voxel size of 0.4 mm. Images were post-processed and analyzed using ROVER (ABX, Radeberg, Germany) and displayed as maximum intensity projections (MIPs) at indicated time points and scaling. The time points displayed in graphs represent the mid-frame timepoints. Three-dimensional volumes of interest (VOIs) were defined using a fixed threshold of 39% for delineation of the tumor. Standardized uptake values (SUV) were determined and reported as SUVmean (VOI-averaged). Time–activity curves were generated for tumor VOIs and further analyzed using Prism (GraphPad Software, San Diego CA, USA).

2.2. Results and Discussion

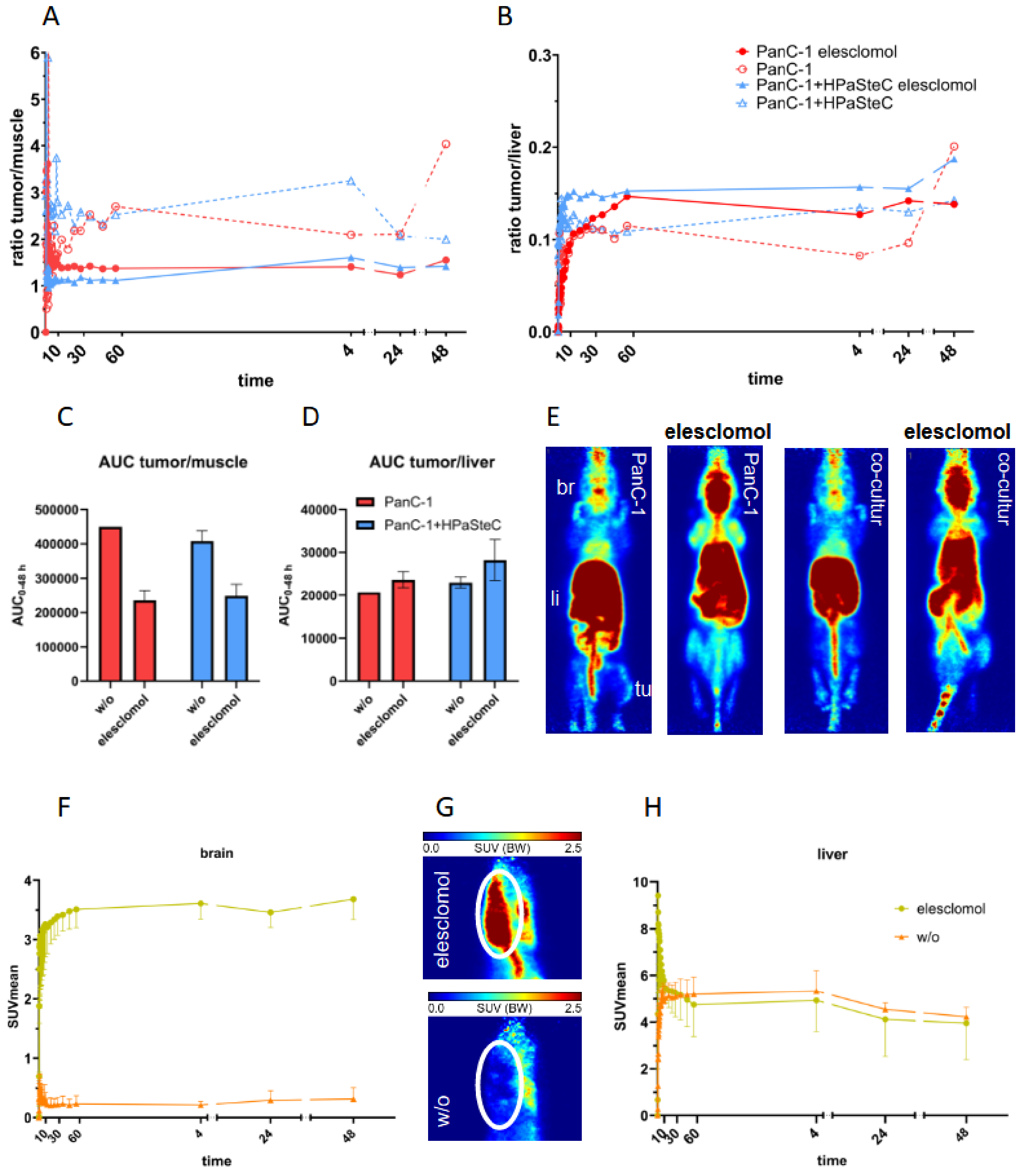

Figure S4: In vivo PET imaging in tumor-bearing mice. Ratio of SUVmean of tumor and muscle (A) and ratio of SUVmean of tumor and liver (B). Area under curve (AUC) of tumor to muscle ratio (C) and tumor to liver ratio (D). (E) Representative MIP images of animals imaged with $[^{64}Cu]CuCl_2$ without ES (left) and with ES (right), indicated position of brain (br), liver (li) and tumor (tu). (F) SUVmean in the brain of mice with or without ES over time. (G) Representative sagittal images of the head of mice injected with or without ES. The white circle indicates the position of the brain. (H) SUVmean in the liver of mice with or without ES over time. In panels A, B, F, and H, the time on the x-axis is presented in 10-, 30-, and 60-minute increments, while the later time points (4-, 24-, and 48-hour) are presented in hours.

For pilot in vivo experiments, ES was selected as it demonstrated the most promising results in cellular experiments in vitro. The in vivo model employed a subcutaneous xenograft model with PanC-1 and a co-culture with PanC-1+HPaSteC. The model used here is described in detail elsewhere [1]. HPaSteC injected alone did not form tumors, as reported elsewhere, and were therefore excluded [1, 2]. The uptake ratio between the tumor and the muscle (Figure S4A) as well as the tumor and the liver (Figure S4B) was calculated and plotted using the

standardized uptake value (SUVmean) obtained from PET experiments. While both ratios are comparable between PanC-1 and co-culture, a higher tumor to muscle ratio was observed in mice with [^{64}Cu]CuCl$_2$ administration alone. The corresponding AUC values supports this finding (Figure S4C+D), demonstrating a diminished tumor-to-muscle ratio in mice injected with ES. Conversely, the tumor-to-liver ratio is enhanced with ES. Figure S4E presents the maximal intensity projection (MIP) of representative mice without and with ES. It can be observed that there is a high ^{64}Cu accumulation in the brain compared to animals imaged without ES (Figure S4G). In contrast, the liver accumulation was high with a SUVmean of 4-5, but comparable between mice with and without ES (Figure S4H). The use of [^{64}Cu]CuCl$_2$ for PET imaging has been demonstrated to be effective for the detection of prostate cancer, bladder cancer, glioblastoma multiforme, and non-small cell lung carcinoma in humans [3]. The suitability of [^{64}Cu]CuCl$_2$ for PDAC was also demonstrated in this preclinical setup. In the subcutaneous tumor xenograft, the tracer uptake over time, as measured by the AUC, was twofold that of the muscle (Figure 6C). Additionally, the findings indicated no notable increase in the accumulation of the radiotracer in combination with ES within the tumors. However, there was an accelerated accumulation of copper within the brains of mice (Figure 6). As previously documented [4], the physiological uptake of copper in the brain is minimal. The elevated levels of ^{64}Cu in the brain following ES administration are presumably attributable to an increase in cytochrome c oxidase levels within the brain and the capacity of ES to traverse the blood-brain barrier [5].

3. In vitro experiments with reference cell line BxPC3

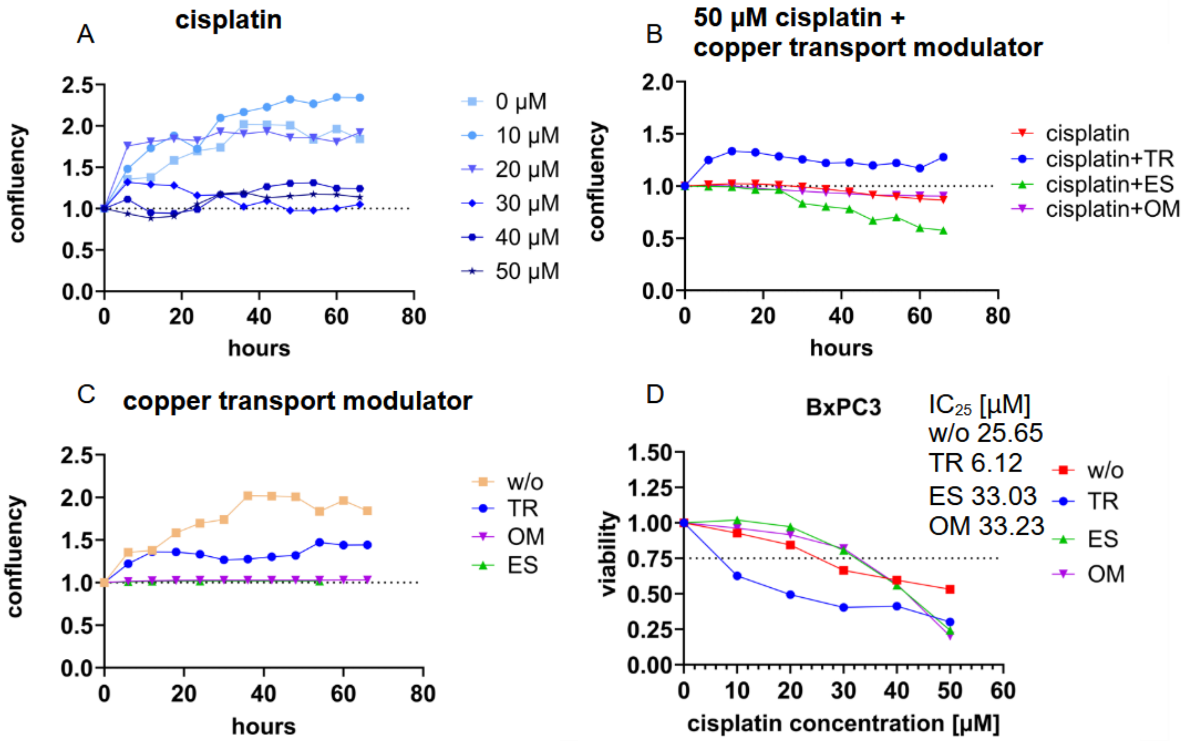

Figure S5: Effect of copper transport modulators and cisplatin on BxPC3 cells. Confluency of BxPC3 (A-C) cells incubated with cisplatin at concentrations 0-50 µM for 66 hours (A). Further, confluency of cell cultures incubated with 50 µM cisplatin and 1500 µM TR, 40 nM ES or 347 µM OM for 66 hours (B) and confluency of cell cultures incubated with 1500 µM TR, 40 nM ES or 347 µM OM for 66 hours (C). (D) Dose-response curves of BxPC3 cells following treatment with only cisplatin or in combination with 1500 µM TR, 40 nM ES or 347 µM OM. Corresponding IC_{25} values calculated for each treatment condition

A stagnation in confluence is observed for BxPC3. BxPC3 cells exhibited a similar degree of confluency to HPaSteC and co-culture cells when incubated with TR and cisplatin in combination, compared to cisplatin alone. The most pronounced reduction in cell confluency of 40% at 66 hours was demonstrated by ES. Subsequently, the effect of TR, ES and OM alone on cell confluency was analyzed. For all cell cultures, we observed an effect on confluence between 12-18 hours after incubation. While untreated cells thrived, growth stagnation was observed for BxPC3. Figure S5D presents the results of the cell viability test, which was conducted at the endpoint, after 66 hours. The change in cell viability is displayed in relation to the concentration of cisplatin. BxPC3 cells exhibited cytotoxic effects for cisplatin and TR as well, but showed also for OM enhanced cytotoxic effects at 50 µM. This is an intriguing finding that is at odds with our previous observations regarding cell confluency in figure S5B. This discrepancy is likely due to the growth characteristics of BxPC3 cells. These cells grow as flat, coherent cell aggregates, which causes dead cells to remain attached, thereby leading to misleading results in confluency studies. Consequently, we anticipate that the viability results depicted in Figures S5D will provide a more accurate representation. Here, we observe an anticancer activity of foremost TR on the cells, reducing the IC_{25} from 25.6 µM to 6.12 µM.

4. Whole Image Western Blots

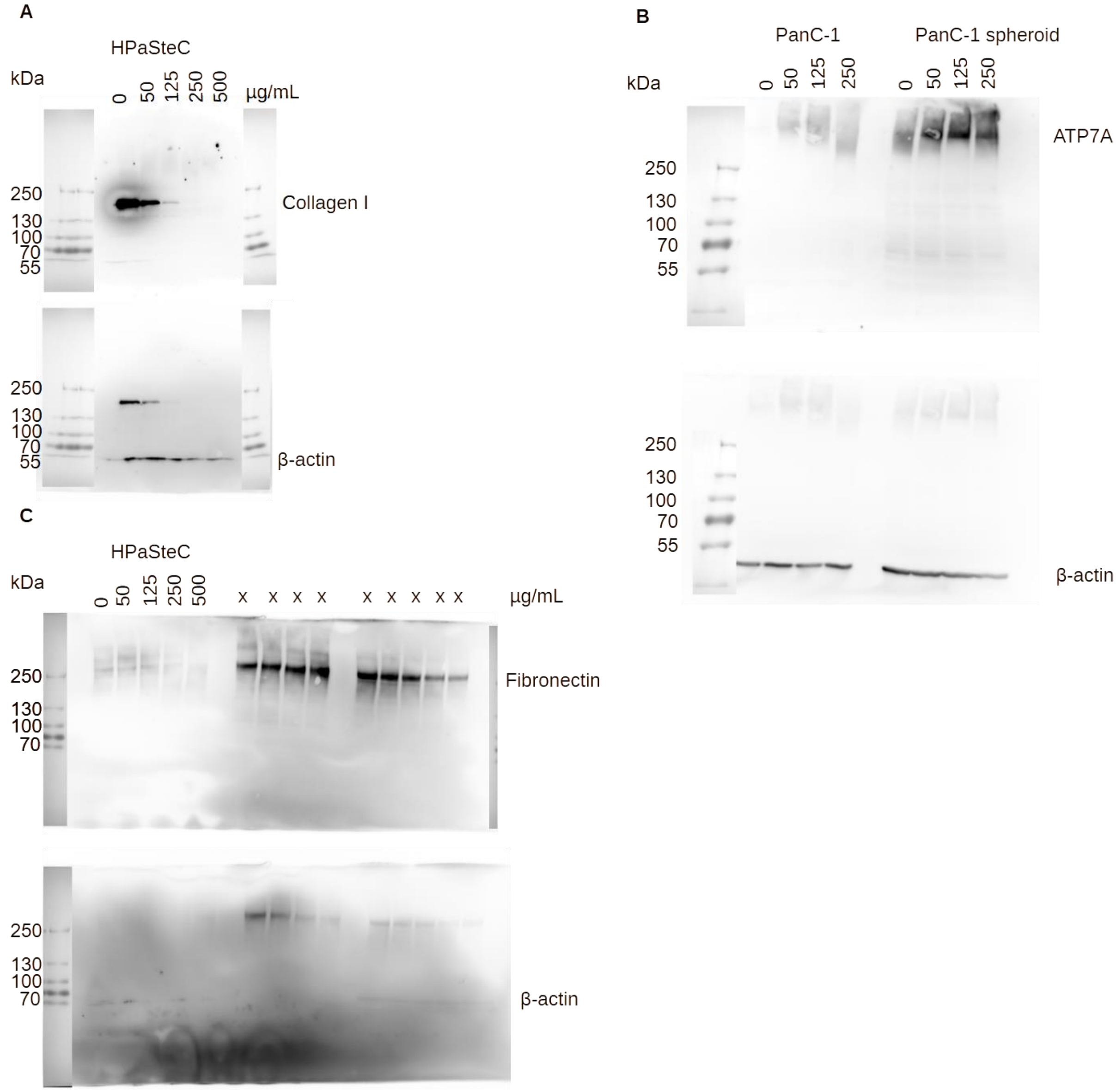

Figure S6: The entire collagen I, ATP7A and fibronectin Western blot image is presented here (A) Western blot against collagen I antibody in HPaSteC cells incubated with tranilast concentrations 0, 50, 125, 250 and 500 µg/mL. (B) Western blot against

ATP7A antibody in PanC-1 cell and spheroid lysate previously incubated with 0, 50, 125 and 250 µg/mL tranilast. (C) Western Blot against fibronectin antibody in HPaSteC cell lysate incubated with 0, 50, 125, 250 and 500 µg/mL Tranilast. All blots with corresponding β-actin.

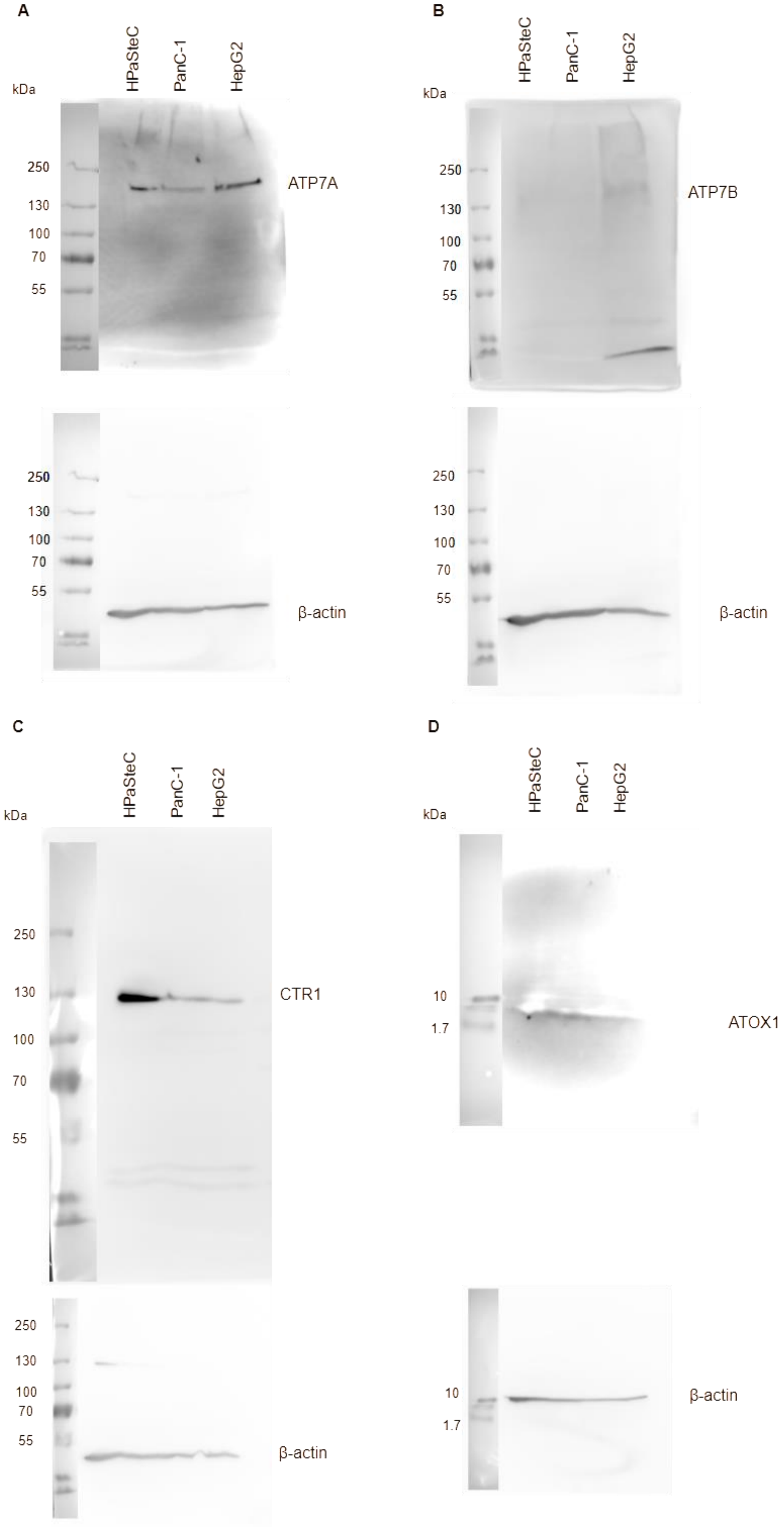

Figure S7: The entire Western blot image with antibodies against ATP7A (A), ATP7B (B), CTR1 (C) and ATOX1 (D) in HPaSteC, PanC-1 and HepG2 cells is presented here. All blots with corresponding β-actin.

5. References

1. Doctor, A., et al., *Combined PET Radiotracer Approach Reveals Insights into Stromal Cell-Induced Metabolic Changes in Pancreatic Cancer In Vitro and In Vivo.* Cancers, 2024. **16**(19): p. 3393.
2. Vonlaufen, A., et al., *Pancreatic Stellate Cells: Partners in Crime with Pancreatic Cancer Cells.* Cancer Research, 2008. **68**(7): p. 2085–2093.
3. Peng, F., *Recent advances in cancer imaging with 64CuCl2 PET/CT.* Nuclear Medicine and Molecular Imaging, 2022. **56**(2): p. 80-85.
4. Peng, F., et al., *Mouse extrahepatic hepatoma detected on MicroPET using copper (II)-64 chloride uptake mediated by endogenous mouse copper transporter 1.* Molecular Imaging and Biology, 2005. **7**: p. 325-329.
5. Guthrie, L.M., et al., *Elesclomol alleviates Menkes pathology and mortality by escorting Cu to cuproenzymes in mice.* Science, 2020. **368**(6491): p. 620-625.
6. Ullrich, M., et al., *Multimodal somatostatin receptor theranostics using [64Cu] Cu-/[177Lu] Lu-DOTA-(Tyr3) octreotate and AN-238 in a mouse pheochromocytoma model.* Theranostics, 2016. **6**(5): p. 650.
7. Ullrich, M., et al., *Epigenetic drugs in somatostatin type 2 receptor radionuclide theranostics and radiation transcriptomics in mouse pheochromocytoma models.* Theranostics, 2023. **13**(1): p. 278.
